# Utilization of a Continuous Pericapsular Nerve Group (PENG) Block with an Opioid-Sparing Repair of a Femoral Neck Fracture in a Pediatric Patient

**DOI:** 10.1155/2020/2516578

**Published:** 2020-07-14

**Authors:** Karla Wyatt, Moustafa Zidane, Chyong-jy Joyce Liu

**Affiliations:** Department of Anesthesiology, Perioperative and Pain Management, Baylor College of Medicine/Texas Children's Hospital, Houston, TX, USA

## Abstract

In the pediatric population, femoral neck fractures are usually associated with high-impact trauma and often present with pain in the groin area. Regional anesthesia can offer adjunctive therapy for acute pain management. Various techniques have been employed to circumvent pain related to hip fractures and resultant hip surgery. Neuraxial, lumbar plexus, caudal, epidural, fascia iliaca, and femoral continuous nerve block techniques are advantageous in mitigating hip pain. However, these approaches require patient repositioning during placement and carry the potential for motor blockade with resultant weakness. A newly described method, the Pericapsular Nerve Group (PENG) block, allows for analgesia of the anterior hip capsule via the obturator, accessory obturator, and femoral nerves while sparing motor blockade. PENG blockade has demonstrated efficacy in both adult and pediatric patients. Herein, we describe the perioperative course of a 9-year-old girl with a transcervical femoral neck fracture who underwent an opioid-sparing open repair with the utilization of a continuous PENG block. PENG blockade via a continuous nerve block resulted in optimal analgesia and markedly reduced perioperative opioid consumption with preserved motor function. Our experience facilitated early discharge and rehabilitation mobility while reducing potential rebound hyperalgesia and enabling parental/patient satisfaction.

## 1. Introduction

Nonpathologic femoral neck fractures in the pediatric population are generally associated with high-impact trauma [1]. Optimal management involves prompt diagnosis, hip stabilization, and open versus closed fixation. Perioperative pain control is of paramount importance following pediatric hip surgery. Inadequate analgesia can contribute to patient and parental dissatisfaction, prolonged recovery, and increased hospital length of stay. While there is a paucity of pediatric data, adult literature has described an association between the utilization of regional anesthesia and decreased postoperative delirium, hospital length of stay, intensive care admission, and overall mortality among geriatric patients undergoing hip replacement surgery [2]. As such, regional anesthesia is advantageous in providing sufficient analgesia while reducing the adverse effects of opioids. Lumbar plexus blockade, neuraxial techniques (caudal or epidural), and combined femoral nerve and fascia iliaca blockade have been shown in a myriad of pediatric studies to exhibit opioid-sparing effects and lower postoperative pain scores in patients who are suffering from hip pain [3–6].

Despite the success of neuraxial techniques in decreasing postoperative pain scores in pediatric patients undergoing hip surgery [4–6], positioning requirements, bilateral sensory and motor blockade, and urinary retention limit their use. Our institutional practice includes administration of a general anesthetic combined with a lumbar plexus continuous nerve block for children undergoing unilateral hip surgery. This effectively blocks the femoral, lateral femoral cutaneous, and obturator nerves which innervate the hip while maintaining functional status on the nonsurgical side, albeit the operative side is still susceptible to motor weakness.

Pericapsular Nerve Group (PENG) block was first described in 2018 by Girón-Arango et al. [7] for perioperative analgesia in hip fracture patients via blockade of the articulating branches of the hip: the accessory obturator nerve (AON), obturator nerve (ON), and the femoral nerve (FN) [7–11]. This technique was confirmed by a cadaveric dye study [12] showing a “true” pericapsular spread that only targeted the sensory branches of the anterior hip capsule with subsequent motor-sparing effects [7]. PENG block was also successfully demonstrated by Mistry et al. whereby five patients with hip fractures all reported pain relief within 15 minutes without resultant quadriceps weakness [13].

Herein, we present the utilization of a continuous PENG block for perioperative pain management in a pediatric patient undergoing hip surgery. To our knowledge, this is the first report describing a continuous PENG block analgesia for a pediatric patient.

Written informed consent was obtained from the parent for this scientific contribution.

## 2. Case Report

**Figure 1:**
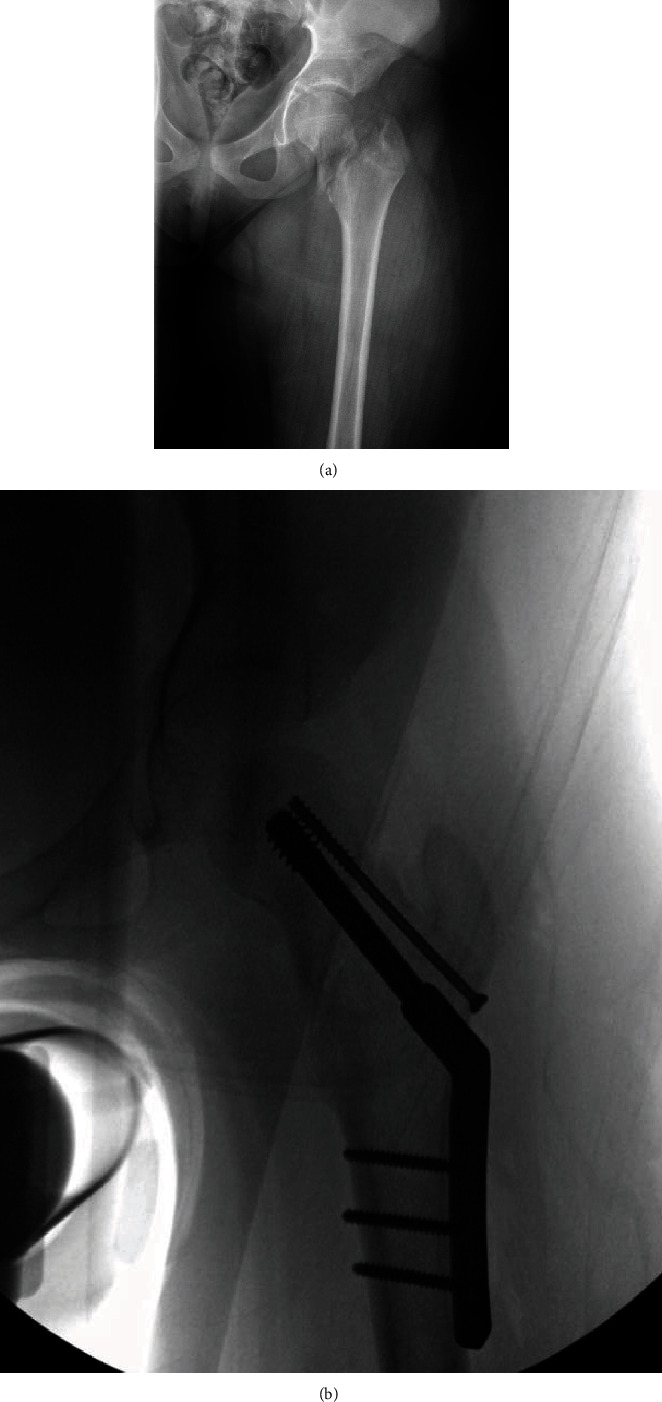
(a) X-ray image of patient's injury; (b) open reduction and internal fixation RIF of intertrochanteric left femur fracture with dynamic hip screw.

A 9-year-old, 45.7 kg, healthy girl presented following a fall from the top of a bounce house with inability to bear weight and hip pain. Imaging revealed a transcervical fracture of the left proximal femur with lateral displacement of the distal femoral segment (Figure 1(a)). There were no distal femoral fractures, intra-abdominal, or spine-related injuries. She was splinted and placed in traction in preparation for an open reduction with internal fixation. Upon discussion with the surgical team, the goals of care included a 2-day admission for postoperative pain control and physical therapy, with complete avoidance of motor blockade. Given the degree of traction, injury-related positioning restraints, and the postoperative motor assessment concern, we offered a PENG block catheter for postoperative pain in lieu of a lumbar plexus or combined femoral and fascia iliaca blockade.

**Figure 2:**
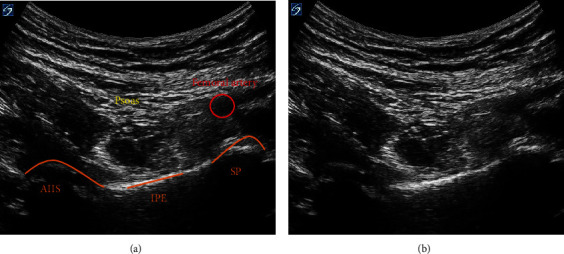
(a) Visualization of the psoas muscle, femoral artery, and anterior inferior iliac spine (AIIS), iliopubic eminence (IPE), and the superior pubic (SP) ramus on sonoanatomy with identification. (b) Sonoanatomy.

**Figure 3:**
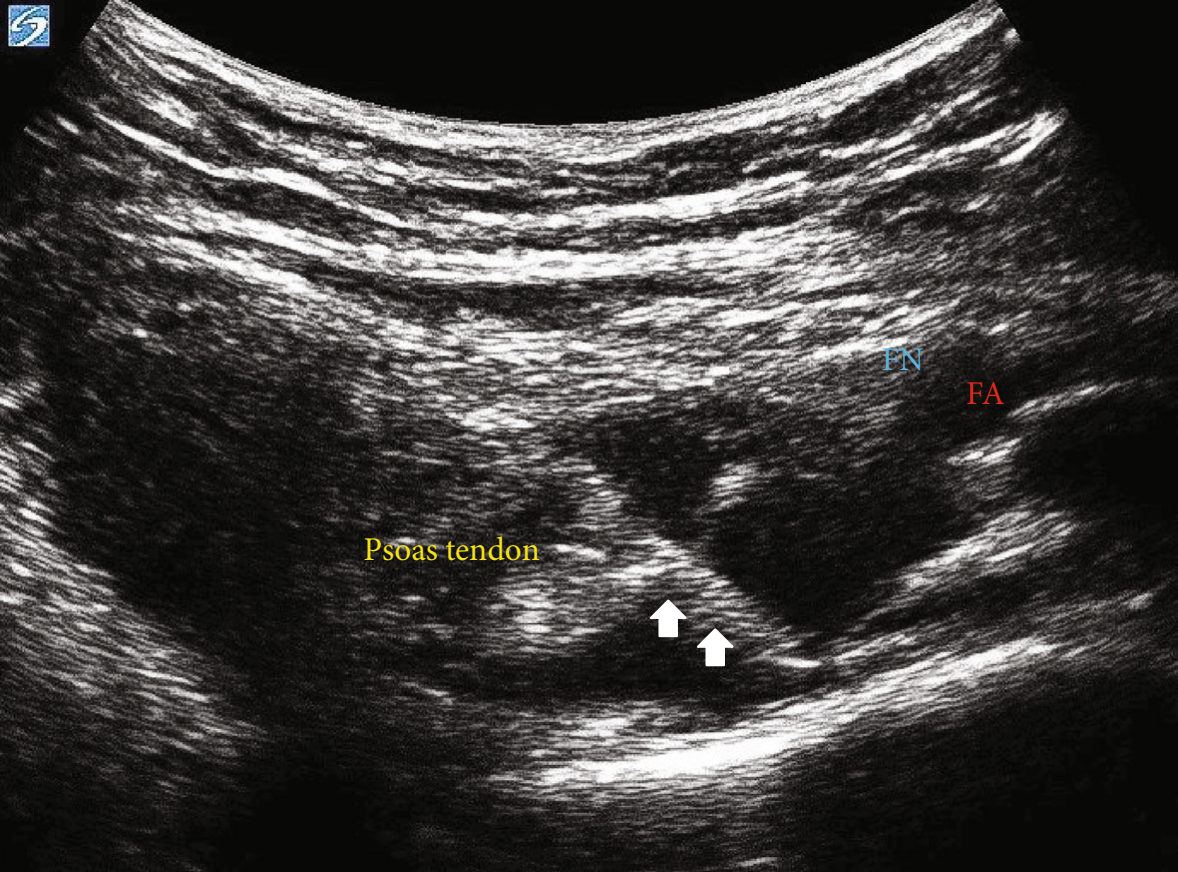
Echogenic needle isolation following contact with the iliopubic eminence. FA: femoral artery; FN: femoral nerve. Arrow represents the needle placement.

Proceeding general anesthesia, the patient was maintained in the supine position with the left lower extremity in traction and satisfactory reduction, confirmed with X-ray imaging. A curvilinear, low-frequency transducer (2-5 MHz) was placed in the transverse plane along the left anterior inferior iliac spine (AIIS) to identify the iliopsoas muscle, femoral artery, and femoral nerve. The probe was then rotated counterclockwise, aligned with the pubic ramus to visualize the AIIS, iliopubic eminence (IPE), femoral artery, psoas muscle, and the superior pubic ramus (Figure 2). Under direct visualization, a 22-gauge (G), 80 mm echogenic needle was advanced in-plane, lateral to medial between the psoas tendon and pubic ramus until the needle made contact with the IPE (Figure 3). The needle was slightly withdrawn, and following negative aspiration, 14 ml of bupivacaine 0.25% (~0.75 mg/kg) was injected in incremental doses. The block performance took 7 minutes. This bolus dose single injection was sufficient for intraoperative surgical analgesia. Preparation and surgical positioning allowed for maximal time for block onset.

While an incision was made along the lateral proximal femur from the vastus ridge distally proceeding to the iliotibial band, there were no changes in heart rate or blood pressure. Following exposure of the lateral surface of the femur, a starting guide pin was placed in the femoral head just short of the physis. The guide pin was used for measurement and placement confirmation prior to the placement of hip screw and plate. The compression and shaft screws were then drilled, measured, and placed through the plate. An additional partially threaded cannulated screw was placed into the neck as an antirotation screw. The final location of implants was confirmed on anterior-posterior and lateral X-ray imaging before irrigation and closure (Figure 1(b)). The patient was maintained on 0.8 MAC of desflurane with no intraoperative opioid requirements. The total operating time was 102 minutes. The procedure was uneventful without any apparent complications. The estimated blood loss was 200 ml.

**Figure 4:**
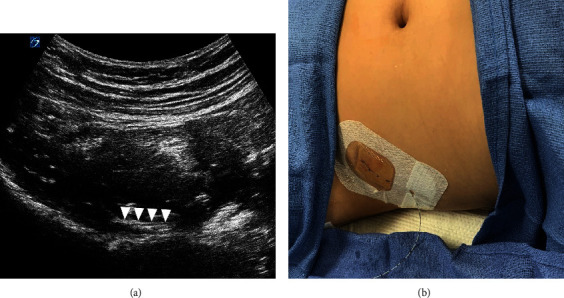
(a) PENG catheterization and corresponding sonoanatomy with isolation of the proximal portion of the peripheral nerve catheter positioned above the iliopubic eminence. Arrow presents the catheter; (b) PENG catheter placement.

A nerve block catheter was placed following surgical repair to avoid location interference with operative access. At the conclusion of the case, image acquisition was again obtained as previously described. Similarly, a 22 G, 80 mm echogenic needle was advanced in-plane (Figure 4). Following lateral to medial advancement, contact with the IPE occurred at a sonographic depth of about 2.5 cm; however, given the lateral approach, the total needle length inserted was 5 cm. An end-hole peripheral nerve catheter was threaded to a depth of 7 cm (2 cm beyond the needle tip) with visualization of the femoral neurovascular structures. Catheter placement was performed in 12 minutes. The procedural anesthesia time was 45 minutes including the induction, emergence, and performance of both regional blocks.

Postoperatively, the patient appeared to be comfortable with numeric pain scores of 2-3/10. The anesthetic infusion via a PENG catheter was initiated with ropivacaine 0.1% at 6 ml/hr (0.13 mg/kg/hr rate). The patient was non-weight-bearing to the left lower extremity per surgical team, but no motor weakness was observed. Physical exam revealed no sensation to light touch on the anteromedial or lateral hip and intact flexion at the knee. Overnight, she did not require any breakthrough opioids or muscle relaxants. FLACC and subjective pain scores were 0/10 for the ensuing 12 hours, and the parents reported good pain control. On postoperative day (POD) 1, the patient was able to participate in physical therapy and gait training with ease. The anesthetic infusion was discontinued on POD 1 (>24 hours since placement of the catheter) after a trial of an oral acetaminophen-opioid preparation. The patient was discharged on the evening of POD1 with as needed diazepam for muscle spasms and an acetaminophen-opioid preparation for pain control. There was no evidence of block complication.

## 3. Discussion

Pediatric hip fractures can be divided into 4 subtypes with type II (transcervical fractures) being the most prevalent, extending through the middle of the femoral neck [1]. Cannulated screw fixation is usually required for older children in whom transcervical fracture is unstable, and the determination of open versus closed approach commonly depends on the success of surgical traction for reduction. Regional anesthetic techniques are safe in children [14–16] and can provide analgesia for pediatric patients undergoing hip surgery [3–6]. Adequate analgesia results in faster rehabilitation, decreased opioid consumption, and avoidance of the untoward side effects associated with opioids.

The single-shot PENG block has recently been described in adult and pediatric literature for perioperative analgesia for hip surgery by blocking the articulating branches of the femoral nerve, accessory obturator nerve (AON), and obturator nerve (ON) [7–11]. Short et al. demonstrated the consistent anatomical contribution of the FN and AON to the anterior hip capsule [11]. The motor-sparing effect and technical ease of image acquisition in the traction-immobilized patient made this blockade ideal for our patient. We also observed similar pain relief in our patient without quadriceps weakness as reported in other studies [12, 13].

A major drawback of the single-shot peripheral nerve blockade is the limited timeframe for analgesic relief. In addition, the potential for rebound hyperalgesia after a single-shot nerve block was described at 12-24 hours [17, 18]. Rebound hyperalgesia is postulated to be due to subjective pain perception or an inadequate pain regimen when single-shot blockade ceases. We would expect the pain associated with major hip surgery to span about 24-72 hours [19]. While additives such as dexamethasone and alpha-2 adrenoreceptor agonists have been documented to prolong the analgesia of single-shot peripheral nerve blocks, their use is off-label and perineural toxicity is lacking in the pediatric literature [19]. Given the potential extent for pain beyond the expected provision of a single-shot nerve block, our decision was to proceed with a continuous infusion via a catheter for this patient.

The use of continuous peripheral nerve blockades in children is safe and efficacious [20–23]. Infusion with optimal concentrations and dosages of local anesthetic can provide sensory analgesia while preserving motor function. Pediatric studies reviewing inpatient and outpatient regimens have replicated decreased opioid consumption with continuous peripheral nerve block and a low incidence of complication [20, 22, 23]. The most common complications cited in children were paresthesias (which resolved with local anesthetic discontinuation), mechanical malfunction, and nausea or vomiting [21]. Given the safety profile of a continuous nerve block and the potential for ongoing postoperative pain, we performed a PENG block for this patient to balance surgical neurovascular and musculoskeletal considerations with optimized regional pain management.

Consistent with the adult literature, our experience demonstrated motor preservation and opioid-sparing analgesia with the utilization of the PENG block. We chose to use the safe and accepted pediatric range of 0.5-1.5 mg/kg bupivacaine for the initial lower extremity bolus dosing with a conservative infusion rate of ~0.13 mg/kg/hr via the catheter [20]. We recognize that the efficacy of the PENG block has not been established with prospective clinical studies. While our observations were promising, future investigation into the efficacy, bolus and infusion rates, and safety of the PENG in the pediatric population is needed.
